# Patterns of patient-reported outcomes (PROs) in a diverse group of gynecologic cancer survivors

**DOI:** 10.1007/s00520-024-08968-4

**Published:** 2024-11-04

**Authors:** Charlotte Gerrity, Abdulrahman Sinno, Akina Natori, Vandana Sookdeo, Jessica MacIntyre, Sophia George, Carmen Calfa, Tracy E. Crane, Frank J. Penedo, Matthew Schlumbrecht

**Affiliations:** 1 Department of Obstetrics, Gynecology, and Reproductive Sciences, University of Miami Miller School of Medicine, Miami, FL USA; 2 Sylvester Comprehensive Cancer Center, 1121 NW 14th St, Suite 345C, Miami, FL 33136 USA; 3 Department of Medicine, University of Miami Miller School of Medicine, Miami, FL USA; 4 Department of Psychology, University of Miami, Miami, FL USA

**Keywords:** Patient-reported outcomes, Gynecologic cancer, Hispanic

## Abstract

**Objectives:**

Racial and ethnic disparities in patient-reported outcomes (PROs) among gynecologic cancer survivors are not well studied. We evaluated whether individual-level characteristics were associated with PROs in diverse gynecologic cancer survivors.

**Methods:**

Gynecologic cancer patients in an ambulatory oncology clinic completed a psychosocial and practical needs assessment before their appointments through the electronic medical record (EMR) patient portal. Assessments were available in English and Spanish. Fatigue, pain, physical function, depression, and anxiety were assessed with Patient-Reported Outcomes Measurement Information System (PROMIS®) computer adaptive tests, and health-related quality of life was assessed by FACT-G7. PROs were dichotomized based on severity (normal/mild vs moderate/severe). Demographic and clinical information was collected. Analyses were performed using Chi-square, t-tests, and Kruskal–Wallis tests.

**Results:**

A total of 582 women completed the assessment; 20% (*n* = 116) were racial minorities, and 54.5% (*n* = 310) were Hispanic. A total of 192 (32.8%) completed the assessments in Spanish. Hispanic patients had lower mean fatigue scores (49.31 vs 51.74, *p* = 0.01), and patients whose preferred language was Spanish had lower mean depression (47.63 vs 48.97, *p* = 0.05) and fatigue scores (48.27 vs 51.27, *p* < 0.01). There were no significant differences in the severity of PROs by race, ethnicity, or preferred language. QOL scores were worse in patients with high symptom severity for anxiety (*p* = 0.04) and physical functioning (*p* < 0.01). Current smokers had worse physical functioning (13.4% vs 6.5%, *p* = 0.03).

**Conclusions:**

We found no significant differences in severity of PROs by race, ethnicity, or preferred language. Quality of life scores were worse for patients with high symptom severity for physical functioning and anxiety.

**Supplementary Information:**

The online version contains supplementary material available at 10.1007/s00520-024-08968-4.

## Introduction

Patient-reported outcomes (PROs) measure symptoms, quality of life, and overall functioning directly from the patient without interpretation by a provider [1]. Routine collection of PROs allows for better patient-provider communication and can facilitate early identification of intolerable symptoms associated with treatment, urgent psychosocial concerns, and other cancer-related complications [2, 3]. This improved identification of patient needs and concerns can guide treatment decisions and connect patients to essential services that may improve survival [2]. Analysis of PROs within specific subpopulations may enhance understanding of the needs and lived experience of cancer survivors of differing disease sites [4–6].

To date, most studies of PROs in gynecologic cancer survivors, and cancer survivors in general, have been conducted in populations with an overwhelming majority of White, English-speaking patients [1–7]. The results of these studies have limited generalizability to clinical settings with more diverse patient populations. Hispanic and Spanish-speaking patients are particularly underrepresented in studies of PROs in patients with gynecologic cancer, yet they represent a growing population in the USA [8]. Philp et al. found that Black and Hispanic patients had less emotional and instrumental support relative to their White counterparts while undergoing evaluation or treatment for gynecologic cancers [6].

Racial and ethnic disparities have been identified in all gynecologic cancers at multiple levels of influence [9–13]. Black women of any ethnicity experience the most profound disparities relative to other races [9, 12, 14]. They have the highest mortality and lowest 5-year survival of all races for cervix cancer regardless of stage at diagnosis or histologic subtype [9]. They also have the highest risk of endometrial cancer of all races and are more likely to present with a high-risk histologic subtype [12, 13]. Black women with endometrial cancer experience more treatment delays, receive less guideline-adherent care, and are less likely to receive referrals to essential supportive care services [6, 12–14]. Hispanic patients are more likely to present with advanced-stage cervix and endometrial cancer [9, 12, 15, 16], less likely to receive standard-of-care treatment for ovarian and endometrial cancer [11, 12, 15–17], more likely to experience treatment delays, and less likely to have surgery at a high-volume surgical center [9, 11, 12, 15–17]. There are also known disparities in health-related quality of life (QOL) and higher unmet supportive care needs among Hispanic and Latino cancer survivors [5, 6, 18–20].

Given known disparities in gynecologic care among racial and ethnic minorities, as well as the lack of data in these populations relative to self-reported symptom burden, our objective was to complete an exploratory analysis to characterize PROs by race, ethnicity, and preferred language in a diverse group of gynecologic cancer survivors and assess their relationships with overall QOL. We hypothesized that PROs would vary by patient demographics and that there would be substantial variation in the context of patient race and ethnicity.

## Methods

### Procedures

In 2019, the ambulatory Gynecologic Oncology clinic at the University of Miami Sylvester Comprehensive Cancer Center (SCCC) implemented a symptom monitoring program through the electronic health record to assess symptoms and needs before appointments and triage patients to supportive care services [7]. Patients presenting for their second or later visit are prompted to complete a wellness assessment called My Wellness Check (MWC) through Epic MyChart before each appointment. Patients are not prompted to complete the evaluation at the first visit, which typically focuses on diagnosis and treatment planning; this visit can create high levels of stress and anxiety that tend to resolve over time. The decision to exclude patients on the first visit was made at the discretion of SCCC leadership. Assessments are available in English and Spanish and take approximately 8–10 min to complete online or in the Epic MyChart patient portal. Patients are prompted to complete the assessment 72 h before each appointment, and patients with frequent appointments are not prompted to complete the survey more than once every 30 days. This research protocol was approved under the University of Miami Institutional Review Board (ID 20220248), with a waiver of consent granted, and was conducted in accordance with the Declaration of Helsinki.

### Questionnaires

The primary measure used for symptom monitoring is Patient-Reported Outcomes Measurement Information System (PROMIS®) Computer Adaptive Tests (CATs), a series of brief, validated symptom questionnaires widely used in ambulatory oncology settings [21–24]. PROMIS® CATs generate T-scores and corresponding severity thresholds (normal, mild, moderate, or severe (Supplement 1) using normative data from patients with cancer and the general population, with 50 being the mean T-score and a standard deviation of 10 in the reference population [7]. Patients are prompted to complete PROMIS® CATs for depression, anxiety, fatigue, pain interference, and physical function. Patients are also reminded to complete the FACT-G7, a seven-item validated questionnaire that assesses the most important symptoms and concerns commonly reported by patients undergoing cancer treatment [25].

Demographic information, including age (continuous), race (White, Black, Other), ethnicity (Hispanic, non-Hispanic), preferred language (English, Spanish), marital status (married, divorced, widowed, single), insurance status (private insurance, government insurance, self-pay), alcohol use (yes/no), and smoking history (never/former vs current), was collected from the EMR. Clinical data, including cancer type (ovary, cervix, endometrium, or other), recurrence (yes/no), and time since diagnosis, were also gathered from the EMR. Stage was excluded as a variable given the significant heterogeneity of the population with regard to disease site, staging schemas, and treatment trajectories.

All patients who completed the MWC PROMIS® CATs between August 2019 and August 2021 were included. The most recently completed questionnaire was included in this analysis for each patient to avoid redundant input from the same patient. Patients who had incomplete PROs (no scores available) were excluded. Patients receiving care at our clinic who did not have a primary gynecologic cancer diagnosis were also excluded.

### Data analyses

Statistical analyses were completed using STATA IC (StataCorp, College Station, TX). Descriptive statistics were used to summarize demographic and clinical characteristics. Hispanic ethnicity and Spanish-speaking were considered as separate variables for all analyses. Given the high rate of bilingualism in Miami-Dade County among Hispanic patients [26], language preference is an inaccurate surrogate of ethnicity and was considered a separate variable. The t-test was used to compare means of raw PRO T-scores across according to patient-level factors. T-scores generated from PROs were dichotomized into two categories by severity threshold: low (normal + mild) vs high (moderate + severe) for comparisons among groups. Chi-square tests (or Fisher’s exact, if indicated) were then used to compare the proportions of high vs low severity responses by demographic factors. The Kruskal–Wallis test was used to compare overall quality of life (QOL) scores according to the dichotomized PROs. All tests were two-sided with significance set at *p* < 0.05.

## Results

During the study interval, 582 women completed at least one assessment. Demographic characteristics are summarized in Table 1. A total of 20% of patients (*n* = 116) were racial minorities, and 54.5% (*n* = 310) were of Hispanic ethnicity. One hundred ninety-two patients (32.8%) completed the assessments in Spanish. Of those women who identified as non-Hispanic, Spanish was the preferred language in 1.9%. Of those who identified as Hispanic, Spanish was the preferred language in 59.4% of respondents (*p* < 0.001). Median age at completion was 56.9 years (range 18.2–92.8). All patients had a confirmed gynecologic cancer diagnosis, and 338 (63.8%) had recurrent disease. The most common cancer type was uterine (*n* = 227, 39.0%), followed by ovary (*n* = 201, 34.5%), cervix (*n* = 98, 16.8%), and other (*n* = 56, 9.7%). Most patients had health insurance (*n* = 562, 96.6%).

**Table 1:** Patient demographics

Demographic information	
*n* = *582 women with confirmed gynecologic cancer*	
Race	*n* (%)
White	458 (80%)
Black	91 (16%)
Other	33 (4%)
Ethnicity	
Non-Hispanic	259 (45.5%)
Hispanic	310 (54.5%)
Preferred language	
English	390 (67.2%)
Spanish	192 (32.8%)
Marital status	
Married	313 (54.9%)
Divorced	98 (17.2%)
Single	122 (21.4%)
Widowed	Widowed 37 (6.5%)
Insurance status	
Private	432 (74.2%)
Government	130 (22.3%)
Self-pay	20 (3.5%)
Alcohol use	
Yes	219 (37.6%)
No	363 (62.4%)
Smoking	
Current/ever	160 (27.5%)
Never	422 (72.5%)
Primary site of disease	
Cervix	98 (16.8%),
Uterus	227 (39.0%)
Ovary	201 (34.5%)
Other	56 (9.7%)
Disease course	
Primary treatment	244 (36.2%)
Recurrence	338 (63.8%)

Raw PRO scores relative to patient characteristics are shown in Tables 2 and 3. Hispanic patients had lower mean fatigue scores than non-Hispanics (49.31 vs 51.74, *p* = 0.01). Relative to patients whose preferred language was English, patients whose preferred language was Spanish had lower mean depression (47.63 vs 48.97, *p* = 0.05) and fatigue scores (48.27 vs 51.27, *p* < 0.01). Older age at diagnosis was associated with higher fatigue scores (*p* = 0.048) but had no association with other PROs. Older age and government insurance coverage were associated with lower physical functioning (*p* < 0.001). Time since diagnosis and age at assessment completion were not associated with any PRO scores. There were no differences in any measures by race or by cancer site. All scores were poorer in patients who had recurred.

**Table 2:** Patient characteristics relative to self-reported scores for anxiety, depression, and fatigue

Variable	Anxiety (mean, SD)	Depression (mean, SD)	Fatigue (mean, SD)
Race			
White	53.19 (9.92)	48.24 (9.87)	50.17 (11.03)
Black	53.16 (9.16)	49.97 (9.59)	50.75 (11.24)
Other	51.77 (10.03)	48 (9.65)	50.8 (9.64)
*p*-value	0.69	0.39	0.81
Ethnicity			
Non-Hispanic	53.00 (9.07)	49.23 (8.92)	51.74 (10.47)
Hispanic	53.44 (10.54)	47.98 (10.65)	49.31 (11.58)
*p*-value	0.51	0.09	0.01
Preferred language			
English	53.07 (9.27)	48.97 (9.28)	51.27 (10.96)
Spanish	53.47 (11.14)	47.63 (11.05)	48.27 (11.06)
*p*-value	0.76	0.05	< 0.01
Marital status			
Married	53 (9.45)	48.52 (9.53)	50.63 (10.89)
Divorced	53.59 (11.21)	47.98 (10.76)	49.92 (11.63)
Single	54 (9.69)	49.60 (9.52)	50.7 (11.19)
Widowed	51.85 (10.59)	48.61 (10.89)	49.61 (11.44)
*p*-value	0.66	0.77	0.89
Insurance			
Private	53.31 (10.10)	48.36 (9.99)	50.01 (11.30)
Government	53.28 (8.96)	49.9 (9.07)	52.43 (10.20)
Self-pay	49.6 (9.23)	44.73 (9.81)	47.05 (9.23)
*p*-value	0.27	0.05	0.08
Disease			
Cervix	53.25 (10.10)	48.29 (9.29)	49.98 (10.53)
Uterine	52.41 (10.04)	48.21 (9.63)	49.22 (10.70)
Ovary	53.44 (9.63)	48.97 (10.13)	51.29 (10.70)
Other	55.12 (9.21)	49.10 (10.63)	52.95 (13.85)
*p*-value	0.26	0.81	0.20
Recurrence			
No	51.61 (10.00)	46.78 (9.53)	48.18 (11.03)
Yes	54.23 (9.59)	49.91 (9.86)	51.77 (10.92)
*p*-value	0.006	0.001	< 0.001

**Table 3:** Patient characteristics relative to physical functioning, quality of life (QOL), and pain scores

Variable	Physical functioning (mean, SD)	QOL score (mean, SD)	Pain (mean, SD)
Race			
White	45.13 (11.38)	19.05 (5.83)	52.35 (10.81)
Black	45.17 (9.86)	18.86 (5.51)	52.40 (9.36)
Other	46.67 (11.36)	19.38 (6.05)	52.62 (10.92)
*p*-value	0.66	0.82	0.99
Ethnicity			
Non-Hispanic	44.97 (10.29)	18.92 (5.52)	51.95 (10.26)
Hispanic	45.31 (12.02)	19.07 (6.01)	52.79 (10.97)
*p*-value	0.87	0.59	0.42
Preferred language			
English	45.41 (10.56)	19.02 (5.67)	52.17 (10.39)
Spanish	44.77 (12.55)	19.11 (6.03)	52.84 (11.07)
*p*-value	0.38	0.71	0.67
Marital status			
Married	45.04 (10.95)	19.16 (5.53)	52.21 (10.33)
Divorced	45.74 (10.90)	19.21 (5.99)	51.24 (10.25)
Single	45.61 (12.02)	18.57 (6.33)	53.85 (11.17)
Widowed	42.94 (11.55)	18.52 (5.89)	53.94 (11.71)
*p*-value	0.81	0.85	0.45
Insurance			
Private	46.03 (11.38)	19.15 (5.89)	52.14 (10.78)
Government	41.41 (9.36)	18.38 (5.51)	53.35 (9.96)
Self-pay	49.21 (12.59)	20.5 (4.58)	50.95 (10.27)
*p*-value	< 0.001	0.23	0.34
Disease			
Cervix	46.11 (11.72)	19.35 (5.54)	53.39 (10.78)
Uterine	45.67 (10.92)	19.34 (6.13)	51.42 (10.93)
Ovary	44.72 (10.74)	18.64 (5.06)	52.97 (9.78)
Other	42.95 (12.83)	18.52 (6.99)	52.28 (11.48)
*p*-value	0.35	0.31	0.25
Recurrence			
No	48.76 (11.30)	20.57 (5.51)	49.88 (10.53)
Yes	42.48 (10.36)	17.90 (5.62)	54.15 (10.30)
*p*-value	< 0.001	< 0.001	< 0.001

There was no difference in the proportion of patients with recurrent disease by ethnicity (non-Hispanic White 62.9% vs Hispanic 63.5%, *p* = 0.88) or primary language (English 64.4% vs Spanish 62.7%, *p* = 0.71). Hispanic patients were significantly younger than non-Hispanic patients (54.6 {SD 12.3} years vs 56.5 {SD 14.0} years, *p* = 0.02), but Spanish-speaking patients were older than English-speaking patients when PROs were reported (61.4 {SD 12.9} years vs 59.1 {SD 13.3} years, *p* = 0.047). There was no difference in age at completion of PROs by ethnicity (non-Hispanics 60.3 {SD 12.9} years vs Hispanic 59.4 {SD 13.4} years, *p* = 0.52). Time since cancer diagnosis at completion of PROs was shorter for Hispanic (non-Hispanic 5.1 {SD 3.1} years vs 3.4 {SD 2.7} years for Hispanic, *p* < 0.001) and Spanish-speaking patients (English preferred 4.40 {SD 2.81} years vs 3.82 {SD 3.29} years for Spanish preferred, *p* < 0.001).

PRO severity was then compared among groups (Table 4). There were no differences in the proportion of patients with high symptom burden for any PRO measures by race, ethnicity, or preferred language. There were also no differences by marital status, insurance coverage, types of disease, or whether the patient had recurred. A higher proportion of current smokers reported worse physical functioning than never/former smokers (13.4% vs 6.5%, *p* = 0.03).

**Table 4:** Proportion of patients with high (moderate/severe) responses by PRO measure (number, %)

Variable	Anxiety	Depression	Fatigue	Physical functioning	Pain
Race					
White	44 (10.6)	48 (12.2)	17 (4.4)	29 (7.4)	18 (4.5)
Black	10 (11.8)	12 (14.5)	2 (2.5)	7 (8.6)	2 (2.5)
Other	2 (9.5)	3 (15)	2 (10.5)	3 (15)	3 (14.3)
*p*-value	0.93	0.81	0.30	0.45	0.07
Ethnicity					
Non-Hispanic	22 (9)	26 (11.2)	12 (5.3)	20 (8.8)	9 (3.4)
Hispanic	34 (12.4)	28 (14.6)	9 (3.8)	19 (7.3)	14 (5.4)
*p*-value	0.22	0.25	0.36	0.56	0.44
Preferred language					
English	35 (9.7)	43 (12.6)	15 (4.5)	30 (8.9)	15 (4.3)
Spanish	22 (13.1)	22 (13.6)	7 (4.4)	10 (6.2)	8 (5.1)
*p*-value	0.24	0.76	0.93	0.30	0.72
Marital status					
Married	32 (11.2)	34 (12.7)	13 (5)	22 (8.3)	12 (4.5)
Divorced	5 (5.6)	10 (11.8)	2 (2.4)	5 (5.9)	1 (1.2)
Single	15 (13.5)	17 (15.9)	5 (4.8)	10 (9.3)	8 (7.5)
Widowed	3 (8.8)	2 (5.9)	1 (3.2)	3 (9.4)	2 (6.1)
*p*-value	0.31	0.48	0.77	0.84	0.22
Insurance					
Private	43 (10.9)	52 (13.9)	18 (4.9)	25 (6.7)	13 (3.4)
Government	12 (10.3)	13 (11.5)	3 (2.7)	12 (10.8)	9 (8.3)
Self-pay	2 (11.1)	0 (0)	1 (6.3)	3 (18.8)	1 (6.3)
*p*-value	0.98	0.24	0.57	0.08	0.09
Disease					
Cervix	9 (10)	10 (11.4)	3 (3.5)	6 (6.7)	3 (3.4)
Uterine	19 (9.2)	29 (14.9)	6 (3.2)	12 (6.4)	7 (3.6)
Ovary	22 (12.0)	20 (11.4)	10 (5.7)	16 (9)	9 (5.1)
Other	7 (13.7)	6 (13)	3 (6.8)	6 (13.3)	4 (8.5)
*p*-value	0.72	0.73	0.55	0.42	0.47
Smoking					
Never/former	32 (11)	32 (11.7)	14 (5.2)	18 (6.5)	13 (4.7)
Current	13 (12.3)	15 (14.7)	3 (3.1)	13 (13.4)	3 (3)
*p*-value	0.73	0.43	0.41	0.03	0.45
Recurrence					
No	18 (10.1)	21 (12.6)	8 (4.9)	11 (6.6)	6 (3.5)
Yes	35 (11.3)	41 (13.9)	13 (4.5)	7 (8.6)	15 (5.8)
*p*-value	0.68	0.69	0.83	0.39	0.43

Overall QOL scores were significantly lower in patients who had high symptom severity for anxiety (*p* = 0.04) and physical functioning (*p* < 0.01) (Table 5). There was no association between overall QOL scores and reported severity for depression, fatigue, and pain. Overall QOL scores did not vary by race (*p* = 0.82), ethnicity (*p* = 0.59), or preferred language (*p* = 0.71).

**Table 5:** Quality of life scores as related to severity of PROs

PRO measure	Mean T-score (SD): low severity	Mean T-score (SD): high severity	*p*-value
Anxiety	19.19 (5.73)	17.39 (5.63)	0.04
Depression	18.95 (5.79)	19 (5.16)	0.99
Fatigue	19.10 (5.66)	17.89 (5.36)	0.31
Physical functioning	19.25 (5.72)	17.02 (4.81)	< 0.01
Pain	18.99 (5.76)	18.93 (5.89)	0.93

## Discussion

Prior studies have reported that racial and ethnic minority patients with cancer have had higher rates of depression and anxiety and overall worse QOL [5, 6, 18–20]. Our cohort, in contrast, demonstrated no differences in QOL or severity of PROMIS scores by race, ethnicity, or preferred language, disproving our primary hypothesis.

QOL scores were significantly lower in patients who had high symptom severity for anxiety and physical functioning. This is consistent with previously reported data which demonstrated that frailty and poor physical function are associated with worse QOL outcomes for cancer survivors in general [27, 28]. Among gynecologic cancer survivors specifically, a high prevalence of anxiety has also been reported [29, 30], with younger cancer survivors and those with poor physical health experiencing relatively higher levels of anxiety [30, 31]. Certain subsets of gynecologic cancer survivors are at further increased risk for poor physical functioning, including smokers [32, 33], endometrial cancer survivors with obesity [34], and patients who have lower extremity edema due to radical surgery and/or lymph node dissection [35]. Patients in these at-risk groups may benefit from targeted interventions aimed at improving physical functioning, including exercise an-d lifestyle interventions, which have been noted to improve both mental health and physical functioning in patients with cancer [36].

We also observed that patients who were current smokers had worse overall QOL scores relative to never or former smokers. Patients with cancer who smoke experience more pain [37], higher cancer-related morbidity [25, 33], and greater treatment-related toxicity, especially when receiving radiation [32, 38]. All of these factors may contribute to frailty and worse physical function. For all cancer types, sustained smoking cessation is associated with lower depression, fatigue, and pain scores over time [39], and there is an inverse relationship between pain severity and years since quitting for former smokers [37]. Support for smoking cessation should be an essential part of comprehensive cancer care for gynecologic cancer survivors, particularly among cervical cancer survivors, who have a high prevalence of smoking and significant treatment-related complications when smoking persists during treatment [32].

Significant differences in the raw PROMIS scores were noted across groups, including lower mean fatigue scores for Hispanic- and Spanish-speaking patients and lower mean depression scores for Spanish-speaking patients. While we are unable to interpret the clinical relevance of these small, but statistically significant variations, these trends are intriguing, and consideration should be given to why these observations were made. One lens through which to evaluate these findings could be the “Hispanic Paradox,” or the observation that Hispanic patients often have equal or better health outcomes compared to non-Hispanic counterparts despite known disparities in access to care [40]. Many cultural and socioeconomic factors have been posited to contribute to the Hispanic paradox, including the “healthy immigrant effect,” which describes the phenomenon of better health outcomes among patients who recently immigrated [40]. This phenomenon is particularly salient for the population served at our institution, as 54% of the population of Miami-Dade County was foreign-born in 2023 [41]. Other cultural and institutional factors, such as provider diversity and a high proportion of Spanish-speaking providers, may account for at least part of the better scores measured in this cohort. Provider-patient ethnic and language concordance in cancer care have been previously associated with improved patient satisfaction, mental health, and medical understanding [18, 42–44]. These factors, however, are not well studied in the context of QOL for patients with gynecologic cancer.

There are several identified weaknesses in this study. It was conducted at a single, private institution. This is a population of almost entirely insured patients receiving care at a high-volume tertiary care center. As such, findings in this population may not be generalizable to uninsured patients in more resource-limited settings. This was also a heterogeneous population, including patients with multiple gynecologic cancer types, which will likely affect results. Our findings were not stratified by cancer type, treatment goals (curative versus palliative), or stage at diagnosis. The exclusion of these data points limits a more granular assessment of the patient experience. Capturing these data is an essential consideration for future investigations when a larger sample size is available and robust multivariable analyses more feasible. Our center has a large Haitian population with many patients who prefer Haitian Creole. These patients were unable to be included in this study as the instruments used have not been validated in Haitian Creole. As such, this study cannot give insight into health-related quality of life in this group which contributes significantly to the Black population in our region. These surveys were offered electronically through a web browser or the Epic MyChart patient portal. This may limit accessibility to patients with low literacy or low familiarity, comfort, or access to these platforms. Finally, we did not evaluate interventions performed before or after these assessments. MWC responses, which are visible immediately to the clinical care team, may prompt interventions (such as referrals to social work, nutrition, and physical therapy) that impact the trajectory of future responses. Similarly, prior supportive interventions which may impact responses were not assessed but could be impacting patient quality of life and therefore measured responses.

Despite these weaknesses, however, to our knowledge, this is the largest cohort of Hispanic and Spanish-speaking patients in any published prospective study of PROs for patients with gynecologic cancer. Racial and ethnic minorities made up over half of the cohort of this study. This is also the most extensive study of this kind to offer PRO surveys in both English and Spanish, and thus the impact of language barriers on data collection was significantly reduced. We postulate that this significant diversity is likely what accounts for the lack of differences in measured PRO severity across all racial and ethnic groups. With such a large population of immigrants, in a city where multiple languages are commonly spoken, and with multiple ethnicities and nativities represented—not just in the general population but also among the physicians and staff—the cancer experience for our patients is likely unique. Our institutional cohort continues to expand, and it is anticipated that future, more granular PRO and QOL data in our gynecologic cancer subpopulations, as well as our racial and ethnic minority groups, will provide clearer evidence about the impact of patient ancestry or sociocultural background on these measures.

## Conclusions

 In a large cohort of diverse gynecologic cancer survivors, race, ethnicity, and preferred language were not associated with symptom severity for any PROs or with overall QOL scores. Higher symptom severity for anxiety and physical function were associated with worse overall QOL, and current smokers were more likely to report worse physical function. Our evaluation of raw PRO scores noted a trend towards lower mean depression, anxiety, and fatigue scores for Hispanic and Spanish-speaking patients. This warrants further investigation in a larger population to determine if such trends translate into significant patient impact during cancer treatment and beyond. Supportive programs, such as those aimed at smoking cessation, routine screening for anxiety and depression, and interventions to improve physical function, are possible means of improving QOL and reducing symptom burden for gynecologic cancer survivors. Such programs should acknowledge how the intersectionality of ethnicity may impact the patient experience and further quantify best practices for the diverse populations of women with these diseases.

## Supplementary Information

Below is the link to the electronic supplementary material.

Supplementary file1 (DOCX 20 KB)

Supplementary file2 (DOCX 19 KB)

## Data Availability

No datasets were generated or analysed during the current study.
