# Supplementary material for: Patterns of patient-reported outcomes (PROs) in a diverse group of gynecologic cancer survivors
Source: Support Care Cancer. 2024 Nov 4;32(11):771. doi: 10.1007/s00520-024-08968-4 (PMC11535011; doi:10.1007/s00520-024-08968-4)
Supplement: Supplementary file 1 — Supplementary file1 (DOCX 20 KB) [file 520_2024_8968_MOESM1_ESM.docx]

**Supplement 1. Dichotomization of PROMIS CATs by severity**

| **Symptom** | **Normal + Mild** | **Moderate + Severe** |
| --- | --- | --- |
| Pain | < 70 | ≥ 70 |
| Fatigue | < 70 | ≥ 70 |
| Depression | < 60 | ≥ 60 |
| Anxiety | < 65 | ≥ 65 |
| Physical Function | > 30 | ≤ 30 |
