# Supplementary material for: Patterns of patient-reported outcomes (PROs) in a diverse group of gynecologic cancer survivors
Source: Support Care Cancer. 2024 Nov 4;32(11):771. doi: 10.1007/s00520-024-08968-4 (PMC11535011; doi:10.1007/s00520-024-08968-4)
Supplement: Supplementary file 2 — Supplementary file2 (DOCX 19 KB) [file 520_2024_8968_MOESM2_ESM.docx]

**Supplement 2**: FACT G7. FACT G7: We assess health-related Quality of Life using FACT G7. FACT G7 is designed to quickly and effectively capture the most relevant issues to cancer patients. There are 7 questions assessing physical, emotional, functional well-being:

1. I have a lack of energy.
2. I have pain.
3. I have nausea.
4. I worry that my condition will get worse.
5. I am sleeping well.
6. I am able to enjoy life.
7. I am content with the quality of my life right now
